# Human endogenous retroviruses and autism spectrum disorder: Brief review of recent literature

**DOI:** 10.1192/j.eurpsy.2023.1939

**Published:** 2023-07-19

**Authors:** R. Mota Freitas, D. Reis Gomes, F. Silveira

**Affiliations:** Department of Psychiatry and Mental Health, Hospital do Espírito Santo de Évora, Évora, Portugal

## Abstract

**Introduction:**

Human endogenous retroviruses (HERVs) are genetic elements resulting from ancestral infection of germline cells. HERVs have been associated with multiple complex disorders, including neurodevelopment disorders, namely autism spectrum disorder (ASD).

**Objectives:**

In this review, we aim to explore the relationship between endogenous retroviruses and autism spectrum disorder.

**Methods:**

A non-systematic review of literature published in the Pubmed database in the last ten years was performed. A combination of the search terms “autism spectrum disorder”, “ASD”, “endogenous retrovirus”, “human endogenous retrovirus”, “ERV” and “HERV” was used. Articles were selected based on title and abstract review.

**Results:**

Preclinical and human studies suggest that the abnormal expression of endogenous retroviruses can represent a biological trait of neurodevelopment disorders in affected individuals and their parents. The precise epigenetic processes that underpin this relationship remain elusive. Nonetheless, HERVs play various roles, modulate host immune response and may affect human embryogenesis. All of these factors can participate in the interplay among genetic vulnerability, environmental risk factors, and maternal immune activation that contribute for the development of ASD.

**Conclusions:**

There is a recent, mounting body of evidence linking HERVs and ASD. Whether HERVs behave as a cofactor for the development of ASD or an epiphenomenon of neurodevelopmental disorders remains unclear. Further research is needed to assess if there is causality and evaluate the potential for HERVs to serve as biomarkers for ASD.

**Disclosure of Interest:**

None Declared

